# Extent of magnitude representation deficit and relationship with arithmetic skills in children with 22q11.2DS

**DOI:** 10.1186/s13023-024-03263-1

**Published:** 2024-07-03

**Authors:** Emilie Favre, Margot Piveteau, Marie-Noelle Babinet, Caroline Demily

**Affiliations:** 1 Laboratoire EMC, Département de Sciences Cognitives, Psychologie Cognitive et Neuropsychologie, Université Lyon 2, Lyon, France; 2 Service de Neuropédiatrie, Hôpital Nord Ouest, Villefranche-Sur-Saône, France; 3 CRMR GénoPsy-Lyon, Centre d’Excellence Autisme iMIND & Pôle HU-ADIS, CH le Vinatier, Lyon, France; 4 CRMR GénoPsy-Lyon, Centre d’Excellence Autisme iMIND & Pôle HU-ADIS, CH le Vinatier, CNRS & Université Lyon 1, Lyon, France

**Keywords:** 22q11.2 deletion syndrome, Arithmetical skills, Magnitude comparison task, Working memory

## Abstract

**Background:**

Previous studies have produced conflicting results concerning the extent of magnitude representation deficit and its relationship with arithmetic achievement in children with 22q11.2 deletion syndrome. More specifically, it remains unclear whether deficits are restricted to visuospatial content or are more general and whether they could explain arithmetical impairment.

**Methods:**

Fifteen 5- to 12-year-old children with 22q11.2 deletion syndrome and 23 age-matched healthy controls performed a non-symbolic magnitude comparison task. Depending on the trial, participants had to compare stimuli with high or low visuospatial load (visuospatial stimuli or temporal sequence of visual stimuli). The participants also completed a battery of arithmetic skills (ZAREKI-R) and a battery of global cognitive functioning (WISC-V or WPPSI-IV), from which working memory and visuospatial indices were derived.

**Results:**

Children with 22q11.2DS responded as fast as healthy controls did but received fewer correct responses, irrespective of visuospatial load. In addition, their performance in the non-symbolic magnitude comparison task did not correlate with the ZAREKI total score, while the working memory index did.

**Conclusion:**

Children with 22q11.2DS might suffer from a global magnitude representation deficit rather than a specific deficit due to visuospatial load. However, this deficit alone does not seem to be related to arithmetic achievement. Working memory might be a better concern of interest in favoring arithmetic skills in patients with 22q11.2 deletion syndrome.

**Trial registration:**

Clinicaltrials, NCT04373226. Registered 16 September 2020.

## Background

22q11.2 deletion syndrome (22q11.2DS) is a genetic neurodevelopmental disorder that is responsible for a wide range of symptoms, including cognitive dysfunction and learning impairment. Previous studies have reported visuospatial [1–3], temporal perception or processing [4, 5], number comparison and, more broadly, arithmetic difficulties that seem unrelated to general cognitive abilities [6–8]. From these data, Simon proposed that spatiotemporal mental representations of patients with 22q11DS might have a coarser resolution. This might thus explain why difficulty in representing time, space, and numerical quantities leads to arithmetic learning disabilities in children [9]. This attractive “*spatiotemporal hypergranularity*” hypothesis is related to the idea of a shared mechanism for time, space and quantity representation [10]. It also assumes that numerical and arithmetical skills stem from non-symbolic magnitude representation abilities such as the collection of items. The idea is that non-symbolic magnitude representations are gradually refined during childhood and allow children to establish themselves more easily in the symbolic number system [11]. Nevertheless, some empirical data from healthy controls and dyscalculic children failed to support these theoretical findings. Alternative models suggest dual separated symbolic/non-symbolic representations [12, 13] or a progressive differentiation of magnitude representation systems with age [14, 15]. Some evidence has also suggested that arithmetic skills rely on other cognitive mechanisms, such as working memory [16–18] or visuospatial perception [19, 20].

In this context of challenging theorical views, two recent studies specifically investigated non-symbolic magnitude representation to define the extent of impairment in 22q11.2DS. Attout et al. enrolled 22q11.2DS patients aged 5 to 23 years. The participants were compared to two control groups (matched for verbal intelligence and visuospatial abilities) aged 3 to 13 years. Participants underwent several magnitude comparison tasks with varying degrees of visuospatial and temporal requirements. The procedure implied that trials with increasing complexity were introduced progressively throughout the task and that the task was discontinued when a participant performed at chance level. A higher complexity is introduced by a smaller ratio of quantities for comparison. This procedure is thought to quantify the resolution of magnitude representation. 22q11.2DS patients were selectively impaired in tasks involving visuospatial information. This result suggested that deficits in magnitude occurred because of a visuospatial deficit [21]. McCabe et al. used a similar paradigm of magnitude comparison tasks with increasing difficulty but obtained opposite results. In the second study, participants with 22q11.2DS were compared to a control group and a group of patients with other genetic disorders. All 22q11.2DS patients were aged 7 to 16 years, and the intellectual quotient (IQ) was consistently lower than that of the controls. 22q11.2DS patients had higher thresholds for both visuospatial and temporal judgment tasks, suggesting reduced resolution in both spatial and temporal magnitude representations [22]. In brief, the study of Attout et al. accounted for separate magnitude representation systems, while the study of McCabe et al. favored shared mechanisms and global deficits.

These conflicting results might be partly attributed to methodological limitations due to the clinical characteristics of 22q11.2DS. It is indeed a relatively rare condition, with an average IQ within the borderline range [23], and common psychiatric comorbidities [24]. The first confounding factor concerns general cognitive impairment. In the study of McCabe et al., patients had lower IQs than controls did, and their IQs were significantly correlated with performance in several judgment comparison tasks. This raises the question of the exact role of IQ in the pattern of results. In the study of Attout et al., patients and controls were matched according to IQ, but the age ranges were wide and highly different between groups. The age effect was thus neglected, although magnitude representation and arithmetic are likely to evolve during childhood due to brain development and schooling [25]. Therefore, it seems important to consider age when determining the extent of magnitude representation impairment in 22q11.2DS patients. The third limitation is the methodological specificity of the experimental procedure. In both studies, trials were presented from the easiest to the heaviest. This might introduce confusion into the results of patients given the high incidence of attention/hyperactivity deficit disorder (ADHD), sustained attention deficit, and cognitive fatigue in 22q11.2DS patients [24, 26, 27].

Beyond these inconsistent data concerning non-symbolic magnitude representation abilities in 22q11.2DS, its relationship with mathematics achievement is still a matter of debate. Studies have provided conflicting cues. On the one hand, visuospatial and numerical processing at 22q11.2 leads to behavioral patterns that could both be related to posterior parietal cortex dysfunction [28]. Number comparison is also correlated with mental arithmetic operation performance [29], suggesting shared dysfunction. On the other hand, in 6- to 12-year-old children with 22q11.2DS, symbolic magnitude representation deficits remained after controlling for visuospatial working memory capacity [30], suggesting the relative independence of symbolic and non-symbolic processing. These mixed data might also be viewed within the broader context of ability development. Briefly, in healthy children, non-symbolic numerical magnitude representation seems to be less consistently correlated with arithmetic ability than symbolic representation is, especially beyond the age of 6 [31], while some studies have shown a stronger relationship for younger children [32]. Interestingly, both symbolic and non-symbolic training for magnitude processing improved the arithmetic abilities of preschool children, but this effect was significantly greater for symbolic training [12]. It is thus possible that developmental effects contribute to drawing a nuanced picture of the influence of non-symbolic magnitude comparison on arithmetic skills in 22q11.2.DS. Additionally, as previously mentioned, cognitive mechanisms other than magnitude representation could also contribute to arithmetic skills, such as working memory and visual perception. These mechanisms are impaired in children with 22q11.2 [33, 34], which may be a possible alternative explanation for their arithmetic deficit.

The magnitude representation abilities of 22q11.2DS children thus remain unclear. Beyond theoretical implications, better characterizing their profile would be highly beneficial for optimizing children’s care. For example, this approach could help to determine whether magnitude processing training [35, 36] or spatial training [37] are relevant for improving arithmetic skills in 22q11.2DS children or should be avoided. The aim of the present study is thus to test hypotheses about a specific or generalized magnitude representation deficit and to explore whether it could contribute to arithmetic learning disability. It is thought to go beyond the limits and conflicting results of previous studies considering simultaneously the effect of age, global cognitive performance, and potential effects due to ADHD or fatigue. To our knowledge, this is also the first study that simultaneously considers the hypothesis of a non-symbolic magnitude representation deficit among alternative cognitive hypotheses to explain arithmetic difficulties in 22q11.2DS patients.

## Methods

### Participants

Fifteen children with 22q11.2DS confirmed by fluorescence in situ hybridization (22q11.2DS group: 8 females, mean age: 7.83 ± 1.92 years) and 23 healthy controls (CTRL group: 14 females, mean age: 7.05 ± 1.84 years) were enrolled in the present study. Children diagnosed with 22q11.2DS were recruited through the Genopsy Rare Disease Center at “Le Vinatier” Hospital in Lyon. Healthy controls were siblings of patients or were recruited by word of mouth. All the children and their parents provided informed consent for participation in the study.

All participants were aged between 5 and 12 years and reported normal or corrected-to-normal vision. Healthy controls had neither neurodevelopmental disorders according to DSM5 criteria nor a history of psychiatric or neurological symptoms. Except for 22q11.2DS, patients had no medical history known to affect brain function. The patients had neither intellectual disability nor autism spectrum disorders. ADHD was diagnosed in 6 patients, 2 of whom received methylphenidate at the time of testing. Three patients were administered a low dose of antipsychotics due to subclinical psychotic symptoms. All the children were enrolled in the class corresponding to their chronological age. Five children with 22q11.2DS benefited from special educational arrangements.

### Procedure

#### Assessment of global cognitive functioning

Participants completed the WISC-V [38] or WPPSI-IV [39] depending on their age. The data were obtained from tests carried out in routine care less than a year ago or completed at the time of study.

#### Arithmetic ability assessment

Participants aged older than 6 years completed the ZAREKI-R [40], a battery of standardized 11 subtests used to assess numerical and arithmetic abilities in children. It includes counting, quantity estimation, reading and writing numbers, comparison of symbolic numbers, operation on numbers, and problem solving.

#### Simple reaction time task

All participants first completed a simple reaction time task. The stimuli (candy drawings) were displayed from the left or from the right of a central cross-fixation session for 50 ms. The children were instructed to touch the screen on the stimulus side as soon as possible with the index finger of their dominant hand. The response window lasted for 1000 ms. A total of 8 trials were displayed for each side. The task was completed in less than 2 min. The simple reaction time task allowed us to check that the children managed to correctly detect the stimuli and were able to respond by touching the screen. Participation in the study was stopped if the child made more than 2 errors.

#### Magnitude comparison tasks

**Fig. 1:**
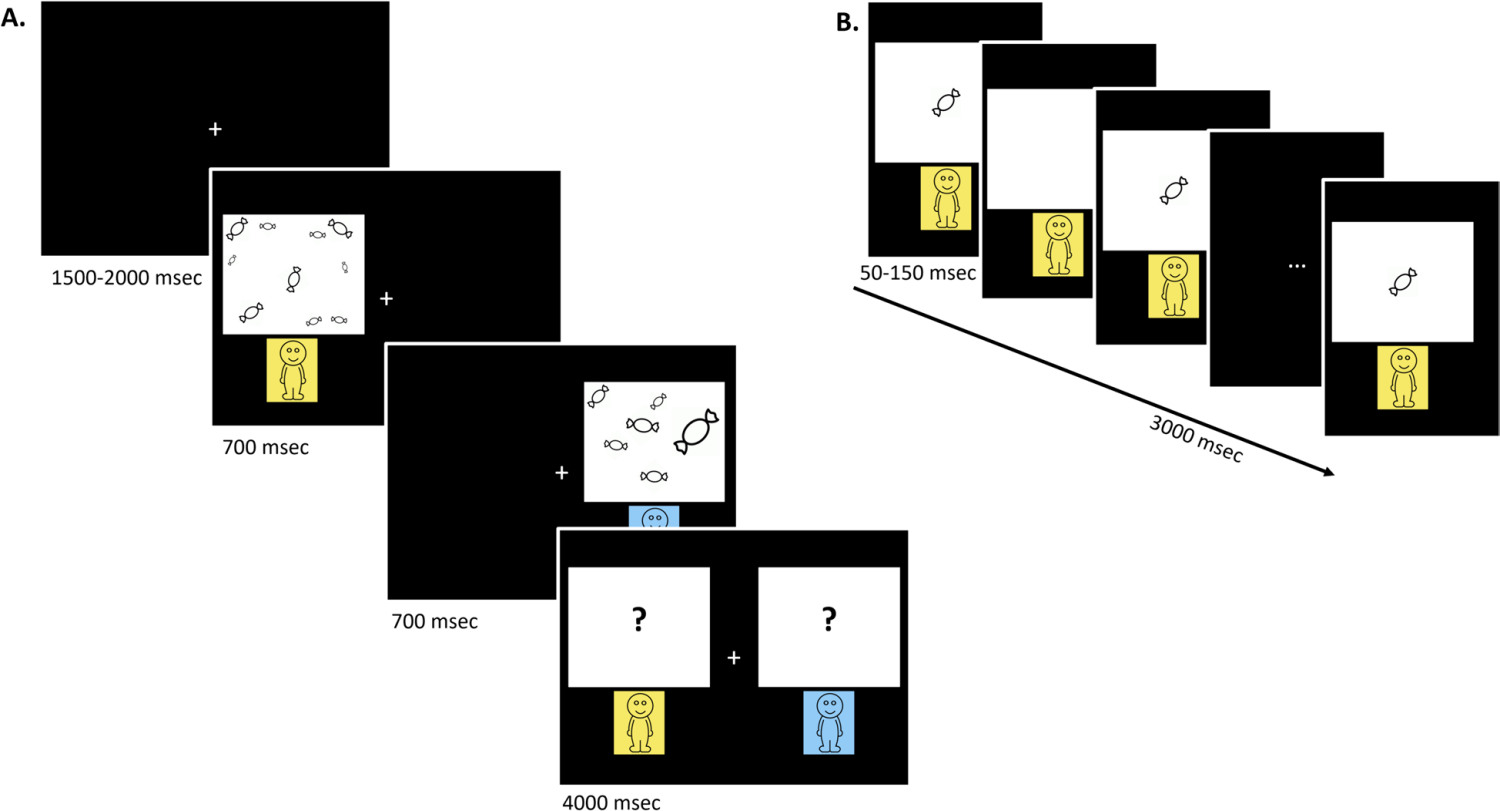
Experimental procedure for the magnitude comparison task. After an intertrial delay, the left- and right-sided stimuli were successively displayed. The children had to choose the figure who had the most candies by touching the right or left side of the screen. The participants were instructed to wait for the response screen with “?” before touching the screen. The participants were encouraged to respond as quickly and correctly as possible.** A** In the V-SPA condition, the stimuli used were visuospatial patterns of candies of varying sizes lasting for 700 ms. **B** In the V-TEMP condition, stimuli were composed of a set of single candies that popped up during 50 to 150 ms. Each stimulus lasted for 3000 ms

The children subsequently completed a magnitude comparison task in which they had to compare two quantities presented successively on one side and the other on the central cross fixation. Immediately after stimuli presentation, the participants had to touch the side on the screen that corresponded to the largest one. The instructions emphasized both speed and accuracy (details are depicted in Fig. 1).

The magnitudes are displayed thanks to non-symbolic stimuli. Depending on the experimental conditions, the stimuli used were visuospatial patterns (V-SPA) or a sequence of visual stimuli (V-TEMP). In the V-SPA condition, the stimuli used were black and white candy drawings of varying sizes and line thicknesses. The difference in the percentage of black ink covering the surface between the stimuli was very small (0.26% on average). In the V-TEMP condition, a black and white candy drawing was drawn several times for 50 to 150 ms. The total duration of candy drawing varied by 5.44% on average between trials.

In both conditions, the magnitude was between 5 and 25 to avoid subitizing effects. The difference between the magnitudes to be compared varied among the 3 different ratios: 1/2, 2/3, and 5/6. Twelve trials are presented as percentages. A larger magnitude was displayed on the right side for half of the trials. The trials were presented in a pseudorandom order: there were no identical pairs in two consecutive trials, no more than three consecutive correct responses on the same side and no more than two identical ratios in succession.

Each experimental condition began with 3 example trials (ratio between stimuli: 1/4). Participation in the study was stopped in case of any error. The order of the conditions was counterbalanced across participants using a Latin square design. All the tasks were completed for approximately 15 min. A break was taken every 5 min. Stimulation and data acquisition procedures were programmed using Presentation 14.8™.

### Data analyses and statistics

Statistical analyses were carried out in JASP (version 0.17.3). Two participants from both groups were discarded from the analyses because they declined to complete the experimental tasks. All the remaining participants had fewer than 2 errors in the simple reaction time task and succeeded in all the training trials of magnitude comparison tasks. The rate of correct responses varied from 0.47 to 0.92. Full-scale intellectual quotient (IQ) scores, working memory index (WMI), and visuospatial index (VSI) were computed with respect to the WISC-V or WPPSI-IV normative sample. Concerning arithmetic abilities, a total score (ZAREKI-R score) and a global z score (ZAREKI-R z score) were derived from normative data of the ZAREKI-R battery. A cutoff score of -1.65 was used to classify children with and without arithmetic impairment. Age, sex, IQ score and ZAREKI-R z score were compared between the two groups via an independent sample t test and the Khi2 test. Concerning the magnitude comparison tasks, the rate of correct responses (CR) and mean reaction time (RT) were considered after excluding trials in which the RT was lower than 150 ms and exceeded 3 standard deviations. To overcome deviation from normality and violation of the equal variance assumption, cubic and log transformations were applied to CR and RT, respectively.

Statistical analyses first focused on group differences in the magnitude comparison task to test the two alternative hypotheses of a specific and a general magnitude representation deficit. To this end, the effect of the magnitude representation was assessed by means of a 2-group (22q11.2DS, CTRL) × 2 condition (V-TEMP, V-SPA) ANOVA with repeated measures. The magnitude representation deficit hypothesis was further tested by measuring the effect of the ratio. It is predicted that children with 22q11.2DS should be specifically impaired at a smaller ratio. The effect of the ratio was thus assessed by means of a 2-group (22q11.2DS, CTRL) × 3 ratio (1/2, 2/3, 5/6) ANOVA with repeated measures. Post hoc analyses were performed with the Holm correction. Second, the influence of potential confounding factors, namely, IQ score and ADHD diagnosis, on the magnitude comparison performance was explored. Because they cannot be considered appropriate covariates [41, 42], Spearman correlation analyses were separately conducted for each group to estimate how IQ scores were associated with CR and RT in the magnitude comparison task. The effect of ADHD on CR and RT in the magnitude comparison task was assessed via the nonparametric Mann‒Whitney test because of the very small sample size. Third, analyses focused on how cognitive abilities are associated with arithmetical skills. Spearman correlations were separately conducted for each group to estimate the extent to which the ZAREKI-R total score was associated with magnitude comparison performance (CT), working memory (WMI), and visuospatial (VSI) performance. The effect of arithmetic learning disability (ZAREKI-R z score < 1.65) on the WMI, VSI, CR and RT in the magnitude comparison task was assessed via the nonparametric Mann‒Whitney test because of the very small sample size.

Given that magnitude comparison and arithmetic abilities are thought to increase during childhood [43], statistical analyses were systematically conducted a second time with age as a covariate.

## Results

### Characteristics of participants

There was no significant difference in the mean age (22q11.2DS group: 103.13 ± 22.39 months *vs*. CTRL group: 91.52 ± 22.42 months, t(34) = 1.53, *p* = 0.14, d’ = 0.52) or sex distribution (22q11.2DS group: 7 females *vs*. CTRL group: 13 females, χ^2^ = 0.52, *p* = 0.47) between groups. The total IQ score was within the normal or borderline range for all participants. Five of the 13 patients obtained an abnormal total score in favor of an arithmetic learning disability. The IQ score (22q11.2DS group: 82.53 ± 10.90 *vs.* CTRL group: 118.14 ± 7.49, t(34) = 11.64, *p* < 0.001, d’ = 0.70) and ZAREKI-R z score (22q11.2DS group: -1.45 ± 2.21 *vs.* CTRL group: 1.44 ± 0.94, t(29) = 4.70, *p* < 0.001, d’ = 0.46) were significantly lower in 22q11.2DS patients than in healthy controls.

### Magnitude representation

CRs were significantly lower and longer RTs were more common in the V-TEMP condition than in the V-SPA condition (CR: F(1,34) = 66.51, *p* < 0.001, ɳ^2^ = 0.35; RT: F(1,34) = 117.71, *p* < 0.001, ɳ^2^ = 0.29). The group effect reached significance for CR (F(1,34) = 8.85, *p* = 0.005, ɳ^2^ = 0.10) but not for RT (F(1,34) < 1, ɳ^2^ = 0.001): the 22q11.2DS group had a lower rate of correct responses than did the CTRL group. The interaction effect between group and condition did not reach significance for CR (F(1,34) < 1, ɳ^2^ < 0.001), while this effect was significant for RT, with a small effect size (F(1,34) = 4.24, *p* = 0.05, ɳ^2^ = 0.01). Nevertheless, post hoc analyses indicated that RT did not significantly differ between groups for each condition (V-TEMP: t = 0.95, *p* = 0.70, d’ = 0.32; V-SPA: t = 0.48, *p* = 0.70, d’ = 0.16).

**Fig. 2:**
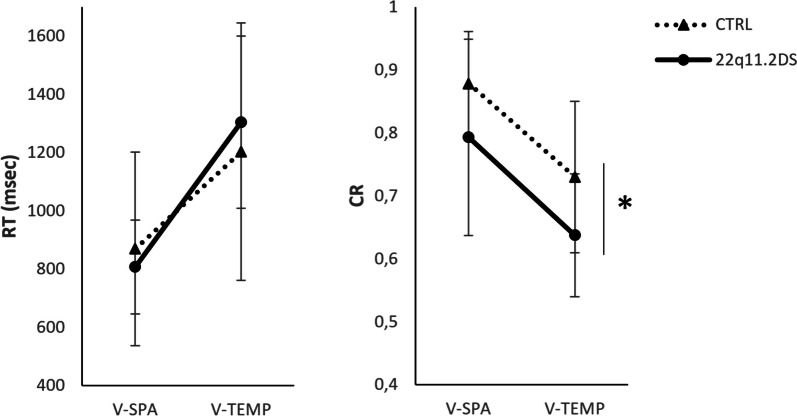
Performance of participants in non-symbolic magnitude comparison tasks. The mean reaction times (RTs) are presented on the left, and the rates of correct responses (CRs) are presented on the right. V-SPA = magnitude comparison task with visuospatial stimuli; V-TEMP = magnitude comparison task with sequence of visual stimuli; CTRL = healthy children; 22q11.2DS = children with 22q11.2 deletion syndrome. * = *p* < 0.001 considering age as a covariate

Considering age as a covariate (CR: F(1,33) = 16.36, *p* < 0.001, ɳ^2^ = 0.17; RT: F(1,33) = 19.44, *p* < 0.001, ɳ^2^ = 0.31), the CR of participants tended to be significantly lower in the V-TEMP condition than in the V-SPA condition (F(1,33) = 3.11, *p* = 0.09, ɳ^2^ = 0.02), while the effect did not reach significance for RT (F(1,33) = 1.47, *p* = 0.24, ɳ^2^ = 0.005). The group effect reached significance, with a large effect on CR (F(1,33) = 20.21, *p* < 0.001; ɳ^2^ = 0.21), but remained nonsignificant for RT (F(1,33) = 2.02, *p* = 0.16, ɳ^2^ = 0.03). Importantly, the effect of group on performance did not interact with magnitude representation (CR: F(1,33) < 1; RT: F(1,34) = 1.28, *p* = 0.27, ɳ^2^ = 0.003; RT: F(1,33) = 2.86, *p* = 0.10, ɳ^2^ = 0.01). In other words, taking account of age, children with 22q11.2DS responded as fast as healthy controls did but received fewer correct responses in both the V-TEMP and V-SPA conditions (Fig. 2).

### Ratio effect

As expected, the analysis revealed that the 22q11.2DS group had a lower CR than the CTRL group did (F(1,34) = 8.58, *p* < 0.01, ɳ^2^ = 0.10). Most importantly, the ratio had a significant effect on CR (F(2, 68) = 40.79, *p* < 0.001, ɳ^2^ = 0.29). Post hoc analysis indicated that the CR was lower for the smallest ratio than for the other two ratios (ratio 5/6: 0.66 ± 0.12, ratio 2/3: 0.82 ± 0.12, ratio 1/2: 0.82 ± 0.14, *p* < 0.001). There was no interaction between group and ratio (*p* = 0.66). No significant effect was detected for RT (*p* > 0.17).

Considering age as a covariate (CR: F(1,33) = 18.57, *p* < 0.001, ɳ^2^ = 0.17; RT: F(1,33) = 17.6, *p* < 0.001, ɳ^2^ = 0.31), the group effect reached significance, with a large effect on CR (F(1,33) = 21.02, *p* < 0.001, ɳ^2^ = 0.20), but remained nonsignificant for RT (F(1,33) = 1.95, *p* = 0.17, ɳ^2^ = 0.03). Neither the ratio effect nor the interaction effect reached significance for CR or RT (*p* > 0.15). In other words, while children with 22q11.2DS overall received fewer correct responses, their pattern of response according to ratio size did not differ from that of healthy controls.

### Role of potential confounding factors

ADHD diagnosis had no significant effect on CR or RT in the magnitude comparison task (CR: W = 12, *p* = 0.39; RT: W = 16, *p* = 0.83). The IQ score did not significantly correlate with CR or RT in the magnitude comparison task in either the 22q11.2DS group (CR: *ρ* = 0.15, *p* = 0.62; RT: *ρ* = -0.29, *p* = 0.33) or the CTRL group (CR: *ρ* = 0.09, *p* = 0.72; RT: ρ 0.26, *p* = 0.25). Correlations remained nonsignificant with partial-out age (22q11.2DS group, CR: *ρ* = -0.26, *p* = 0.42 and RT: *ρ* = -0.36, *p* = 0.26; CTRL group, CR: *ρ* = 0.17, *p* = 0.49 and RT: *ρ* = -0.24, *p* = 0.32).

### Relationship between arithmetic skills and cognitive abilities

In both the 22q11.2DS group and the CTRL group, the ZAREKI-R score was not correlated with the WMI, VSI, or RT and CR on the comparison judgment task (*p* > 0.10). Interestingly, after excluding age, the ZAREKI-R score of the 22q11.2DS group was significantly correlated with the WMI (*ρ* = 0.65, *p* = 0.02) but not with the VSI (*ρ* = 0.40, *p* = 0.19), CR (*ρ* = -0.08, *p* = 0.80) or RT (ρ = -0.15, *p* = 0.65). In the CTRL group, the ZAREKI-R score was significantly correlated with CR (*ρ* = 0.54, *p* = 0.02) but not with RT (*ρ* = 0.17, *p* = 0.51), the VSI (*ρ* = -0.30, *p* = 0.25) or the WMI (*ρ* = 0.31, *p* = 0.23). In other words, taking account of age, the arithmetic skills of healthy controls correlated with comparison judgment task performance, while the arithmetic skills of 22q11.2DS patients correlated with working memory performance. Additionally, in the 22q11.2DS group, an arithmetic learning disability had no significant effect on performance in the magnitude comparison task (CR: W = 21, *p* = 0.94; RT: W = 21, *p* = 0.94) or VSI (W = 9, *p* = 0.12), contrary to WMI. Children with arithmetic learning disabilities tended to have lower WMIs than did other children (ZAREKI-R z score lower than -1.65: 78.2 ± 6.02; ZAREKI-R z score higher than -1.65: 91.3 ± 11.7; W = 7, *p* = 0.07).

## Discussion

The present study assessed the magnitude representation abilities of children with 22q11.2DS to determine whether their impairment was limited to visuospatial stimuli or more generalized. It also aims to explore to what extent this could contribute to their arithmetical skills. Participants thus completed a non-symbolic magnitude comparison task with conditions that varied the visuospatial load. The participants also completed two validated tests to assess their cognitive abilities and arithmetic skills. As previously indicated, the experimental procedure and statistical analyses were designed to address further limitations of previous studies that found conflicting results. Additionally, it is worth noting that a number of clues point to the fact that the experimental task used in the present study indeed allows us to capture the magnitude representation abilities of participants. All of them were able to detect stimuli since they succeeded in completing the simple reaction time task. Moreover, floor and ceiling effects were ruled out since all participants performed above chance and no one received 100% correct responses. Significantly, a ratio effect was found—indicating that the smaller the ratio was, the lower the performance—as classically reported in magnitude comparison tasks [44]. The fact that it was no longer captured after controlling for age is highly consistent with the increase in magnitude representation abilities during childhood [43]. Performance was also lower for sequences of visual stimuli than for visuospatial patterns, as previously reported with a similar paradigm [21, 22].

The results indicated that, taking into account the effect of age, children with 22q11.2DS had fewer correct responses than controls did in the non-symbolic magnitude comparison task. Importantly, this effect was found in all the experimental conditions, that is, irrespective of visuospatial load. Thus, these findings point toward a generalized magnitude comparison deficit, as suggested by several previous studies [4, 22, 30], and contradict the idea of a specific impairment due to the visuospatial dimension [21]. Divergence from the results of Attout et al. could be driven by controlling for age effects and an experimental design that makes non-symbolic tasks with and without visuospatial load more directly comparable to each other. Indeed, in the study of Attout et al., stimuli with high visuospatial loads that must be compared are simultaneously displayed, while others are successively displayed on the screen. This approach might make difficult to compare tasks with each other and to reach conclusions on specific impairments due to 22q11.2DS.

Nevertheless, the present study did not provide strong evidence to support the ‘*hypogranularity’* hypothesis [9] since the ratio effect in children with 22q11.2DS did not significantly differ from that in healthy children. Three nonmutually exclusive hypotheses can be proposed to explain this phenomenon. The first one is in favor of the Simon hypothesis but supports that the ratio effect should not be captured because of unsuitable calibration of the experimental task. Following this idea, children with 22q11.2DS would have coarser resolution that impairs magnitude comparison even at the largest ratio. The second argues that specific impairments in the smallest ratio previously reported [21, 22] are due to the adaptative design of tasks and confounding factors such as fatigue or sustained attention rather than magnitude comparison abilities. These confounding factors are unlikely to contribute to the results of the present study since the ratios were randomly displayed and because ADHD diagnosis seems to not significantly contribute to explaining the variation in non-symbolic magnitude comparison performance. The third explanatory hypothesis relies on global cognitive impairment. According to this view, the performance of patients in the present study was driven by a global cognitive deficit rather than a specific magnitude comparison deficit. The present study provided some clues against this idea. Indeed, patients with 22q11.2DS had fewer correct responses than healthy controls did, but neither group differed according to response time after controlling for age. Moreover, the IQ score was not correlated with performance in non-symbolic judgment comparison tasks. However, we cannot rule out the possibility that global cognitive performance plays a significant role in the differences between groups since they were not matched according to IQ. The lack of an appropriate matching group remains a tricky methodological issue for understanding cognitive abilities in individuals with neurodevelopmental disorders [42]. To address this issue, several cognitive abilities should be tested simultaneously in a larger sample size to determine whether magnitude comparison represents a relative weakness in the cognitive profile of children with 22q11.2DS. In the same vein, it would have been interesting to have a larger sample to be able to describe more precisely the developmental trajectories of magnitude representation acquisition in 22q11.2DS. Previous works have indicated that parietal cortex and executive function development in 22q11.2DS patients follow atypical trajectories and are qualitatively different from those in healthy controls [45, 46]. This is all the more interesting, as these brain areas and cognitive functions are closely linked to number processing and learning abilities.

The second purpose of the study was to investigate the extent to which the non-symbolic magnitude comparison abilities of children with 22q11.2DS are related to arithmetic skills. Although it is commonly thought that both abilities are linked to each other, several studies have failed to clearly corroborate this idea [31]. Interestingly, the present study revealed dissimilar and specific patterns of correlation between arithmetic skills and cognitive functioning according to participant groups. In healthy controls, the ZAREKI-R score correlated with non-symbolic magnitude comparison performance, while it correlated with working memory abilities in patients with 22q11.2DS. Moreover, additional analyses revealed that patients with arithmetic learning disabilities tended to exhibit lower working memory performance than did those with arithmetic skills in the normal range. In contrast, the patient subgroups did not differ according to whether they performed non-symbolic magnitude comparison tasks. Once again, one cannot exclude the possibility that the present results may be limited by the small sample size. However, this pattern is not surprising because arithmetic learning also requires cognitive abilities, such as working memory [16, 47], which are known to be impaired in children with 22q11.2DS [48]. This idea is in line with theoretical accounts that state that, in some cases, dyscalculia can be due to working memory deficits [49] and improved by working memory training [50]. This finding contradicts the findings of a previous study that was specifically interested in the relationship between working memory and arithmetic skills in children with 22q11.2DS. This study revealed arithmetic impairment in patients while they performed as controls in working memory tasks [6]. Arithmetic skills are assessed via separate subtests rather than a composite score, as is the case in the present study. This approach can prevent the capture of global effects. Moreover, it is possible that the population studied was very specific since, surprisingly, patients performed better than controls in certain verbal working memory tasks. Other studies failed to replicate this effect [51, 52]. Regardless of the exact role of working memory, extrapolation of the present results would suggest that promoting non-symbolic magnitude comparison abilities might not be sufficient to favor arithmetic skills in 22q11.2.

## Conclusion

Children with 22q11.2DS might suffer from a global magnitude representation deficit rather than a specific deficit due to visuospatial load. Longitudinal studies could be useful for better capturing developmental trajectories of magnitude representation in this population. However, this deficit alone does not seem to directly explain the arithmetic skills of children with 22q11.2DS. In clinical practice, non-symbolic magnitude representation might not be a prime target for increasing arithmetic skills. Further studies could more directly test the effect of working memory training on the development of arithmetic skills in 22q11.2DS patients.

## Data Availability

The datasets used and/or analyses during the current study are available from the corresponding author upon reasonable request.

## Abbreviations

22q11.2DS: 22Q11.2 deletion syndrome
ADHD: Attention/hyperactivity deficit disorder
CR: Rate of correct responses
CTRL: Control group of healthy children
IQ: Intellectual quotient
RT: Mean reaction times
V-SPA task: Visuospatial magnitude comparison condition
V-TEMP task: Magnitude comparison condition with sequence of visual stimuli
